# Prophylactic Pelvic Drainage of Extraperitoneal Rectal Anastomoses: A Friend or a Foe?

**DOI:** 10.7759/cureus.83905

**Published:** 2025-05-11

**Authors:** Iraklis Perysinakis, Paraskevi Karona, Vasilis Christodoulou, Dimosthenis Michelakis, Evangelia E Vassalou, Eelco De Bree

**Affiliations:** 1 Department of Surgical Oncology, University Hospital of Heraklion, Heraklion, GRC; 2 Department of Medical Imaging, University Hospital of Heraklion, Heraklion, GRC

**Keywords:** anastomotic leakage, extraperitoneal anastomosis, low anterior resection, prophylactic drainage, rectal surgery

## Abstract

The role of prophylactic drain placement after rectal resections with extraperitoneal anastomosis remains unclear and controversial. A systematic search was conducted using the PubMed database for randomized controlled trials (RCTs) and non-randomized controlled clinical trials (CCTs) comparing clinical outcomes of patients with drained and undrained extraperitoneal colorectal or coloanal anastomoses. The primary outcome was anastomotic leakage (AL), whereas secondary outcomes included the impact of drainage on mortality, postoperative bowel obstruction, wound infection, reoperation rate, and length of hospital stay.

Three RCTs and four CCTs that met the inclusion criteria were identified. Two RCTs were judged at a low risk of bias and one at unclear risk. Among CCTs, three were considered to be of fair and one of good methodological quality. Significant discrepancies were encountered among these studies in terms of study design, definition of endpoints, population characteristics, and several technical aspects that render patients’ grouping for conclusion extraction at least demanding, if not unsafe.

In conclusion, there is not sufficient evidence to support routine drainage after rectal surgery with extraperitoneal anastomosis, although drain insertion does not appear to increase postoperative morbidity.

## Introduction and background

Introduction and background

Since their initial utilization dating back to the 19th century during gynecological surgeries, prophylactic abdominal drains have been increasingly used for gastrointestinal (GI) procedures [1]. In this regard, many surgeons have adopted the notion that drainage of the abdominal cavity following GI surgery is linked to improved outcomes. This is highlighted by the fact that the dogma “when in doubt, drain” is still being taught to young surgeons, ignoring the risk of misinterpretation, as to consider drainage as a substitute for a sound anastomotic technique.

Prophylactic drains’ utilization after GI procedures is thought to serve two purposes; it prevents postoperative fluid accumulation and allows timely diagnosis of hemorrhage and anastomotic dehiscence. However, these benefits have to be weighed against the potential drain-related complications, including hemorrhage during placement through the abdominal wall, tissue erosion (hemorrhage, anastomotic dehiscence, or hollow viscus perforation), incisional hernias, postoperative superficial and deep wound infection, foreign body reactions, small bowel obstruction, increased postoperative pain, and prolonged hospital stay [2-4]. Despite the potentially negative impact of routine drain insertion on the surgical outcome, its efficiency in achieving the aforementioned goals has to be taken into consideration.

During the last five decades, the role of prophylactic drainage after various GI procedures has been questioned [3]. Given the considerable ongoing progress in surgery and interventional radiology, the number of randomized controlled trials (RCTs) designed to address this question is constantly rising. Notably, this debate has been extended in recent literature to major complex procedures, such as pancreatectomies, for which omitting prophylactic drainage was traditionally considered inconceivable. Regarding colorectal surgery, there is unanimity in the literature about the futility of routine drain placement after elective colectomies and ileo-colonic, colo-colonic, and intraperitoneal colorectal anastomoses [5,6]. On the contrary, there is an ongoing debate regarding the use of drains after low anterior resection (LAR) with extraperitoneal colorectal anastomoses, which have been linked to a considerably higher rate of anastomotic leakage (AL), owing to the lack of supportive serosal covering. Additionally, drains may prevent the expected fluid collection in the pelvis. However, very few studies have focused exclusively on extraperitoneal anastomoses, whereas in the majority of them, patients with both extraperitoneal and intraperitoneal colorectal anastomoses have been included so as to increase the sample size, at the inevitable cost of a biased comparison in terms of AL. 

This systematic review focuses on the literature regarding prophylactic drainage after elective colorectal resections with extraperitoneal anastomosis, aiming to provide insights into the role of routine abdominal drainage and its impact on patients’ outcome.

## Review

Methods

This systematic review was conducted according to the Preferred Reporting Items for Systematic reviews and Meta-Analyses (PRISMA) guidelines and registered with the International Prospective Register of Systematic Reviews (PROSPERO: CRD420251007929) [7]. Permission for the study was obtained from an Institutional Review Board.

Study Outcomes

The primary objective was to summarize the existing evidence regarding the impact of prophylactic drainage on (clinical and radiological) AL after coloanal, ileoanal, and low colorectal anastomoses.

Secondary objectives included the impact of drainage on mortality, postoperative bowel obstruction (PBO), wound infection, reoperation rate, and length of hospital stay (LOS).

Study Identification

We carried out a comprehensive unrestricted search of the literature for relevant studies up to 31 December 2024, using PubMed (http://www.ncbi.nlm.nih.gov/pubmed). The following keywords were used in the search: ((prophylactic drainage) OR (routine drainage) OR (drain)) AND ((colorectal anastomosis) OR (low anterior resection) OR (extraperitoneal colorectal anastomosis) OR (coloanal anastomosis) OR (ileoanal anastomosis) OR (infraperitoneal colorectal anastomosis) OR (pelvic colorectal anastomosis)). The search was restricted to articles published in English. Moreover, the references of reviewed articles were scrutinized to obtain any other references that eluded the primary search.

Selection Process and Eligibility Criteria

The search was independently conducted manually by two reviewers (IP and PK). All identified studies were evaluated for inclusion using the following eligibility criteria. In order to be included, studies should report at least the primary or one of the secondary outcomes of the present study. The results were compared, and any discrepancies were resolved by consensus after consulting a third reviewer (EdB). 

For sensitivity reasons, only RCTs, prospective and retrospective controlled clinical trials (CCTs) regarding the role of prophylactic drainage in extraperitoneal colorectal/coloanal anastomoses were included in this review. Studies reporting outcomes of both intra- and extraperitoneal rectal anastomoses were included as long as subgroup analysis of patients with extraperitoneal anastomoses was available. 

Exclusion criteria were as follows: studies without comparison of drained and undrained anastomoses; studies reporting outcomes of intraperitoneal colorectal anastomoses; non-human studies; editorials; comments; letters; reviews; meta-analyses; case reports and case series.

Study Risk of Bias Assessment

Methodological quality of the included studies (risk of bias) was assessed by two independent reviewers (IP and PK) using the Jadad scale for RCTs and the Newcastle-Ottawa scale for non-randomized controlled trials [8,9].

Data Collection Process

Clinical appraisal and data extraction were conducted independently by two reviewers (IP and PK) after reading the full text for all articles included. Any discrepancies were resolved by consensus after discussion with a third reviewer (EdB). The following information was extracted from each article, using a predefined data sheet: study identifier (first author, publication year), study design, type of anastomosis, number of patients, drain type, duration of drainage, diverting stoma, definition of AL, duration of follow-up/endpoint for removal, and clinical outcomes (AL, mortality, PBO, wound infection, reoperation rate and LOS).

Results

Search Results

**Figure 1:**
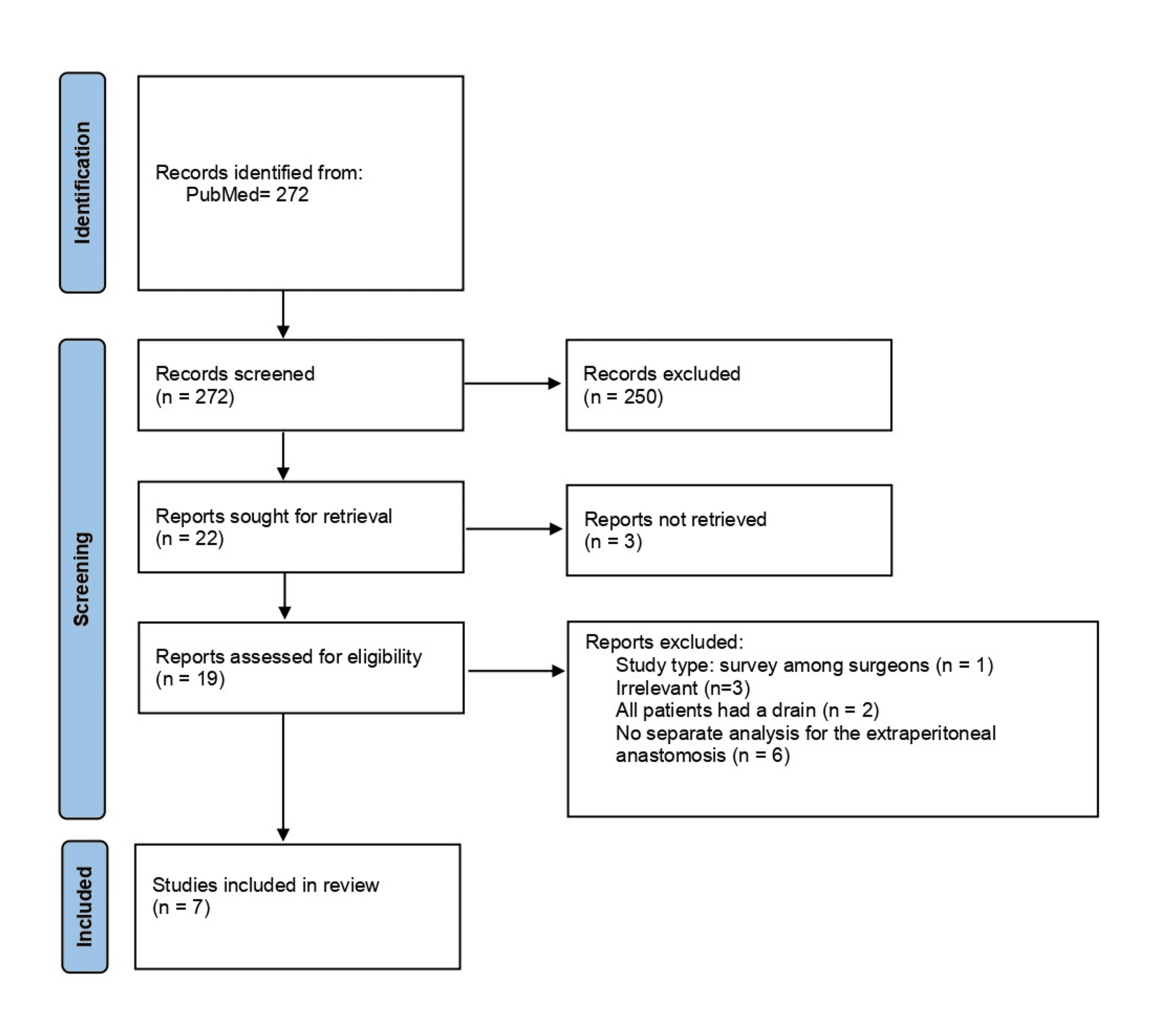
PRISMA 2020 Flow Diagram PRISMA 2020 flow diagram describing the selection process of articles included in this systematic review PRISMA: Preferred Reporting Items for Systematic Reviews and Meta-Analyses

Literature search using the aforementioned criteria identified three RCTs and four retrospective CCTs evaluating the significance of pelvic drainage after extraperitoneal rectal or anal anastomoses [10-16]. The Preferred Reporting Items for Systematic Reviews and Meta-Analyses (PRISMA) flow diagram in Figure 1 describes the article selection process in detail. Table 1 summarizes the main characteristics and results of the seven studies that were included herein.

**Table 1:** Studies Reporting on Prophylactic Drainage of Extraperitoneal Rectal Anastomoses Main characteristics and results of seven studies regarding the impact of prophylactic pelvic drainage on the outcome of patients with extraperitoneal rectal or colo-/ileoanal anastomoses. RCT: randomized controlled trial; CCT: non-randomized controlled clinical trial;  †: both intra- and extraperitoneal rectal anastomoses; ‡: the number corresponds to the subgroup of patients with extraperitoneal anastomoses, not the entire study population; §: deep surgical site infection; ||: drain policy (includes both drained and undrained patients); ¶: no drain policy (includes both drained and undrained patients); NA: not available; AL: anastomotic leakage; PBO: postoperative bowel obstruction; LOS: length of hospital stay; IPAA: ileal pouch anal anastomosis

		Merad et al. [10]	Brown et al. [11]	Denost et al. [12]	Matsuda et al. [15]	Lee et al. [14]	Luberto et al. [13]	Crippa et al. [16]
Year		1999	2001	2017	2018	2021	2023	2024
Study design		RCT	RCT	RCT	CCT	CCT	CCT	CCT
Level of anastomosis		All ^†^	extraperitoneal	extraperitoneal	extraperitoneal	extraperitoneal	extraperitoneal (pouch surgery)	extraperitoneal
Patients		132 ^‡^	60	469	200	996	97	272
Drain		63	31	236	110	551	46	84 ^||^
No drain		69	28	233	90	445	51	188 ^¶^
Type of pelvic drain		suction	suction	suction	suction	suction	capillary	suction or capillary
Duration of drainage		≥5 days	≥2 days or <100ml serosanguinous	when clear output and <100mL	3 days	NA	NA	NA
Diversion		NA	discretion	discretion	all	discretion	discretion (93/97)	discretion
AL, %	Drain	12.7	NA	14.8	NA	14.4	6.5	10.7
	No drain	11.6		15.1		8	5.9	11.2
Clinical AL, %	Drain	NA	7	NA	11.8	NA	NA	NA
	No drain		7		10			
Radiological AL, %	Drain	NA	3	NA	10	NA	NA	NA
	No drain		11		11.1			
Pelvic sepsis, %	Drain	NA	NA	16	12.7	NA	NA	NA
	No drain			18	10			
Mortality, %	Drain	NA	3	NA	0	NA	NA	NA
	No drain		3		0			
PBO, %	Drain	NA	10	19.9	9.1	NA	13	NA
	No drain		3	13.9	8.9		11.8	
Wound infection,%	Drain	NA	16	NA	NA	3.3 ^§^	NA	NA
	No drain		11			0.8 ^§^		
LOS, days	Drain	NA	7	12.2	NA	NA	5	6
	No drain		7.5	12.2			5	5
Reoperation, %	Drain	NA	NA	16.6	NA	12.1	NA	NA
	No drain			21		10		

Quality Assessment

Two out of the three RCTs reported adequate random sequence generation and allocation concealment [10,11]. In the GRECCAR 5 study [12], the vague description of the randomization process (“performed by the surgeon the day before the surgery after obtaining the patient’s written informed consent”) and the absence of any statement regarding allocation concealment suggest an unclear risk of selection bias. Moreover, none of these studies performed blinding of patients, personnel and outcome assessors [17]. The trials by Merad et al. and Brown et al. were judged at a low risk of bias (Jadad score 3), whereas the GRECCAR 5 at unclear risk (Jadad score 2). Among non-randomized CCTs, three were deemed of fair quality and one was deemed of good quality, based on the Newcastle-Ottawa scale. Details regarding quality assessment of CCTs are presented in Table 2. 

**Table 2:** Quality Assessment of Non-Randomized Controlled Trials Included Based on the Newcastle-Ottawa Scoring System ^a^An overall score of seven to nine stars is considered as good quality (low risk of bias), four to six as fair quality (unclear risk of bias) and three or less as poor quality (high risk of bias)

First author	Selection				Comparability	Exposure			Total score^ a^
	Adequate case definition	Representativeness of the cases	Selection of Controls	Definition of Controls		Ascertainment of exposure	Same method of ascertainment for cases and controls	Non-response rate	
Matsuda et al. [15]	*	*		*		*	*	*	6
Lee et al. [14]		*		*	**	*	*	*	7
Luberto et al. [13]		*		*	*	*	*	*	6
Crippa et al. [16]	*	*		*		*	*	*	6

Comparability of Studies

There are also significant discrepancies among these studies in terms of study design, definition of endpoints, population characteristics and several technical aspects that render patients’ grouping for conclusion extraction at least demanding, if not unsafe. For example, in the recent retrospective study by Crippa et al. the study population consisted of a Drain Policy group and a No-Drain Policy group, depending, not on whether a drain was used or not, but on the era during which the patient was operated (before or after the implementation of the “no-drain policy”). Consequently, contrary to all previous studies, both groups included drained and undrained patients, aiming to present, according to the authors, real-life data as well as the degree of surgeons’ adherence to the implementation of this protocol [16]. 

Although the correlation of drainage with AL has been set as the primary endpoint for the majority of studies, the definition of AL is not unanimous. In most of the studies, the definition is in agreement with the Italian multi-consensus on the definition and management of AL, according to which a defect of the intestinal wall at the anastomotic site leading to a communication between the intra- and extraluminal compartments diagnosed by surgical procedure, endoscopy or contrast enema is considered as AL. A pelvic abscess close to the anastomosis diagnosed by CT scan, even without any evident communication with the colonic lumen, is also considered as AL [18]. On the contrary, some studies have used a less wide definition of AL. In the RCT by Merad et al., AL was defined as discharge of feces from the drain or at reoperation or autopsy [10]. Other researchers have differentiated AL from pelvic abscess, given that the primary endpoint of their studies was pelvic sepsis, which included AL, pelvic abscess and peritonitis [12,15]. In the multi-institutional study by Lee et al., the authors did not use a uniform definition of AL. Instead, the diagnosis was taken for granted if documented in the medical records based on each individual institution’s diagnostic criteria [14]. Apart from the different definitions of AL, dissimilarity exists among studies also in terms of AL severity. Four studies have evaluated the overall AL rate [10,12,13,16], two have distinguished clinical from radiological (asymptomatic) leaks in the analysis [11,15], whereas Lee et al. excluded asymptomatic leaks from their study [14].

Another poorly defined endpoint is wound infection. Lee et al. have adopted the most widely used definition classifying incisional surgical site infections (SSIs) as superficial SSIs, deep SSIs, and organ/space SSIs (including abscess) [14,19]. On the other hand, Brown et al. simply defined wound infection as “pus coming from the wound” [11]. 

Moreover, the study populations of these seven studies are heterogenous in several aspects. In five out of seven studies, patients were operated for rectal cancer, whereas in two of them, patients with benign diseases were included as well [10,13]. Patients with rectal cancer received neoadjuvant radiotherapy (NRT) as indicated in four studies [12,14-16], in two others there was no data regarding NRT [10,13], whereas in the RCT by Brown et al., patients with NRT were excluded [11]. Finally, all studies have enrolled patients with extraperitoneal rectal anastomoses as well as those with anal anastomoses, with the exception of Luberto et al. who reported data only from patients undergoing ileal pouch-anal anastomosis (IPAA), that comprise a high-risk group for anastomotic failure [13].

Technical characteristics that differ among relevant studies include the type of drainage used, the time of drain removal and the rate of diverting stomas. In two retrospective studies and in all three RCTs, closed suction drains have been used [10-12,14,15]. Luberto et al. reported the combined use of capillary pelvic and transanal drainage in pouch surgery [13]. Duration of drainage has not been specified in the non-RCTs, whereas various criteria for drain removal have been used in the three RCTs, ranging from “at least two days” to “when clear output and less than 100ml”. Regarding diversion ostomy, in most studies, placement of a protective stoma was performed at the surgeon’s discretion, although there are studies in which all or nearly all patients were diverted [13,15]. 

Anastomotic Leakage

The RCT by Merad et al. included patients with both intraperitoneal and extraperitoneal anastomoses. The subgroup with extraperitoneal anastomoses consisted of 132 patients for whom analysis was conducted only in terms of overall AL rate, without significant differences between the drainage and non-drainage groups (12.7% vs 11.6% respectively) [10]. 

Brown et al. included only extraperitoneal anastomoses and distinguished between clinical and radiological AL, showing that both drained and undrained patients had the same clinical AL rate (7%), whereas the radiological AL rate was higher in the non-drainage group (11% vs 3%). According to the authors the differences between groups were not significant. Notably, no specific information regarding the results of statistical analysis is provided in the article [11].

In the third RCT by Denost et al., the primary endpoint was pelvic sepsis within 30 days which was defined as occurrence of AL, pelvic abscess or peritonitis. No significant differences were found between the two arms, either in pelvic sepsis rate or any of its components. Sixteen percent of drained patients developed pelvic sepsis as opposed to 18% of undrained patients. AL, pelvic abscess and peritonitis rates in the drain group were 14.8%, 11.5% and 3.4% respectively, while in the no-drain group, the corresponding rates were 15.1%, 15.2%, and 4.3% (p=0.94, 0.24, and 0.60, respectively) [12]. 

The same primary endpoint was used in a retrospective study by Matsuda et al. in 2018, which also showed that routine drainage does not decrease the risk of pelvic sepsis or AL in patients after LAR [15]. A large retrospective study by Lee et al. including 996 patients reported an increased risk for AL in the drain group on univariate analysis (14% vs 8%, p=0.041). However, no significant association was confirmed on multivariate analysis [14]. Luberto et al. also failed to demonstrate significant benefits from routine drainage in pouch surgery, as the AL rates were comparable between drain and no-drain groups (6.5% vs 5.9%, respectively, p=1.000) [13]. Finally, AL rates were similar between no-drain and drain groups (11.2 vs 10.7%, p=1) in the study by Crippa et al., without any differences in the AL grade between the two groups (p=0.759). As mentioned before, both groups in this study included drained and undrained patients, in different percentages; drains were used in 76.2% of patients in the Drain Policy Group, whereas only in 16.5% in the No-Drain Policy Group, suggesting a good adoption rate of the no-drain policy [16]. 

**Figure 2:**
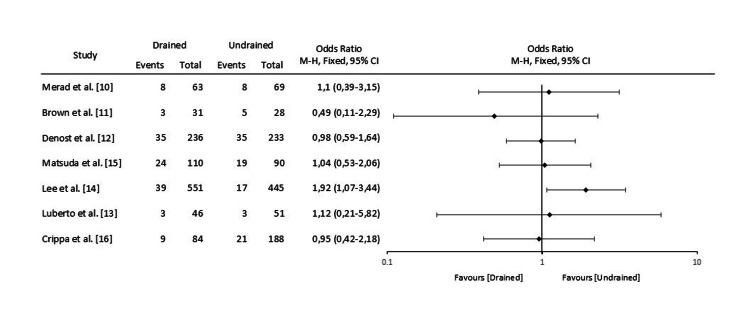
Forest Plot of Odds Ratios for Anastomotic Leakage Between Drained and Undrained Patients With Extraperitoneal Rectal Anastomoses in the Seven Studies Included in This Systematic Review

The Forest plot of odds ratios for AL between drained and undrained patients in the seven studies included in this systematic review is presented in Figure 2.

Mortality

The impact of routine drainage of extraperitoneal anastomoses on mortality has been evaluated in two studies. Brown et al. reported 3% mortality in both arms of the RCT (two patients). One of them died of pulmonary complications and the other died due to myocardial infarction after acute upper gastrointestinal hemorrhage [11]. Moreover, in their retrospective study, Matsuda et al. reported the absence of 30-day postoperative mortality in both groups [15].

Reoperation

The results of the GRECCAR 5 study indicate a higher reoperation rate in undrained compared to drained patients (21% vs 16.6%, respectively). Nevertheless, this difference was not statistically significant (p=0.22) [12]. On the contrary, Lee et al. found a slightly higher risk of 12.1% for reoperation in drained patients compared to a 10% risk in undrained patients, without determined statistically significant difference [14]. 

Length of Stay

The results of two RCTs and two retrospective studies suggest that there is no impact of routine drainage on the duration of hospitalization. Brown et al. reported seven days of hospitalization for drained patients and 7.5 days for undrained patients, with the difference being non-significant (p-value not mentioned) [11]. Denost et al. and Luberto et al. reported exactly the same LOS in both groups of their studies (12.2 and five days respectively) [12,13]. In the study by Crippa et al., the LOS was marginally higher in the Drain Policy Group than in the No-Drain Policy Group, but the difference was not statistically significant (six vs five days, p=0.059) [16].

Postoperative Bowel Obstruction

Brown et al. reported higher incidence of PBO in the drain group (10%) than that in the no-drain group (3%), a difference though that did not reach statistical significance (p-value not mentioned). In accordance with this trial, the results of the GRECCAR 5 trial also suggested a tendency for PBO in the arm with drain (19.9%) compared to the arm without drain (13.9%). Again, this difference was not statistically significant (p=0.08) [11,12]. Moreover, in two retrospective studies, the authors found similar rates of PBO in both drained and undrained patients (9.1% vs 8.9% and 13% vs 11.8%, respectively) [13,15].

Wound Infection

Wound infection has been included as an endpoint in two studies. The results of the RCT by Brown et al. denoted a higher incidence of wound infection in drained patients (16%) compared to undrained patients (11%). However, this difference was not statistically significant (p-value not mentioned) [11]. Lee et al. also reported a trend towards increased risk for deep SSIs in patients with drain, but this association was not confirmed by multivariate analysis [14]. In the GRECCAR 5 trial, although wound infection was documented, the rate was not reported per se but as a component of postoperative morbidity [12]. 

Discussion

This systematic review including three RCTS and four retrospective CCTs supports that prophylactic drainage of extraperitoneal rectal and anal anastomoses neither improves patients’ clinical outcome nor predisposes to drain-related complications. 

The anastomotic level below the peritoneal reflection and especially at a distance of < 5 cm from the anal verge represents a well-established risk factor for AL [20]. This relies on the lack of the supportive peritoneal covering in low-lying anastomoses as well as on the fact that mesorectal dissection results in a large raw, non-peritonealized surface often bearing the detrimental effects of preoperative radiotherapy. Together with the fact that the anastomosis is located in a confined, narrow space, it is reasonable to assume that fluid reabsorption is likely hampered, with potentially negative sequela to anastomotic healing. Moreover, drains are considered a means of early detection of AL, allowing for prompt intervention and improved outcome. However, it is not unusual for the catheter to become clogged by clots, debris or fat, or to be removed before the onset of AL. Thus, the efficacy of drainage in achieving the aforementioned goals is limited. Consequently, contrary to intraperitoneal colorectal anastomoses for which avoidance of drain placement is an evidence-based practice, pelvic drainage after extraperitoneal anastomoses remains an unresolved issue [5,6].

All three RCTs presented in this review have drawn similar conclusions regarding the role of routine drainage in patients with extraperitoneal rectal and anal anastomoses. The multicenter RCT by Merad et al., in which the subgroup of extraperitoneal anastomoses consisted of 132 patients with benign and malignant diseases, concluded that prophylactic drainage does not improve outcome or impacts the severity of complications [10]. The second RCT by Brown et al. included 60 cancer patients and found no difference in morbidity with or without the use of a drain [11]. Finally, the GRECCAR 5 study, the largest RCT with 469 cancer patients, suggested that pelvic drainage is not superior after LAR and should be avoided except in case of bleeding or beyond TME surgery [12].

Further evidence against routine drainage is provided by the four retrospective studies presented herein. In the study by Matsuda et al., no advantages of pelvic drain were demonstrated in patients undergoing laparoscopic LAR with diverting stoma, and drainage did not increase postoperative pain or bowel obstruction. Consequently, the authors recommended against routine placement [15]. According to Lee et al., routine drainage during LAR may not be warranted, as it does not decrease the risk for AL or infectious complications, even in subset analysis of high-risk patients [14]. Moreover, a similar outcome presented by Luberto et al. in patients undergoing IPAA surgery with and without pelvic drainage questions the usefulness of drainage [13]. Finally, the implementation of a No-Drain Policy in 2017 in two tertiary referral colorectal centers in Italy received a good adoption rate by the surgeons and did not affect negatively the surgical outcomes, according to Crippa et al. [16].

It has been suggested that drains represent potential sites of bacteria entry into the abdominal cavity, thus predisposing to SSIs. According to the COMPASS study, intraperitoneal drain insertion after elective colorectal procedures was associated with a 2.5-fold increased risk of SSIs [5]. However, evidence presented herein does not support this hypothesis, as none of the six studies found significantly higher rates of wound infection in drained patients. A possible explanation could be that in all studies, except from the one by Luberto et al., closed suction drains had been utilized, to which lower rates of drain-related septic complications are attributed, compared to other drainage systems.

Drain-related mechanical bowel obstruction has been reported in the literature, mainly in sporadic reports [4]. Potential mechanisms of this complication include twisting of an intestinal loop around the catheter as well as promotion of adhesion formation due to foreign body reaction. Although the results of two RCTs indicated a tendency towards increased risk for PBO in drained patients, statistically significant association was confirmed in neither of them [11,12]. 

Despite heterogeneity, studies regarding the impact of prophylactic drainage on the outcome of patients with extraperitoneal rectal anastomoses have been systematically reviewed and meta-analyzed by four different research groups during the last decade. However, the study selection process regarding the anastomotic level has not been stringent in all meta-analyses.

The first systematic review was published in 2013 by Rondelli et al. and included three RCTs and five non-RCT studies [21]. Although it is clear from the title of the article that the study focuses on extraperitoneal anastomotic drainage, only two out of eight studies included referred exclusively to extraperitoneal anastomoses [10,11]. In one of the RCTs, published by Sagar et al., almost half of the patients had undergone high anterior resection and subgroup analysis of cases with extraperitoneal anastomosis was not conducted [22]. Similarly, all five non-RCT studies referred to a mixed population of patients with both intra- and extraperitoneal anastomoses after anterior resections [23-27].

The results of two subsequent meta-analyses by Guerra et al. [28] and Menahem et al. [29] raised concern regarding the potentially negative impact of drainage on the PBO rate. Pooled data in both studies indicated a significantly higher rate of PBO in drained patients, potentially resembling a drain-related complication. No other significant association was found in either of these studies. However, it has to be stressed that the meta-analysis by Guerra et al., apart from the three RCTs presented here, also included the aforementioned RCT by Sagar et al., thus increasing heterogeneity in the study population in terms of anastomotic height.

Finally, the most recent meta-analysis by Podda et al. showed no benefit from drainage after extraperitoneal rectal anastomosis in terms of AL rate, overall morbidity, wound infection and the need for reintervention [30]. The PBO rate was slightly higher in the drain group, but the difference was again not statistically significant. Notably, the RCT by Sagar et al. was also included in this meta-analysis but the authors performed subgroup analysis of extraperitoneal anastomoses from which this particular RCT was excluded. 

## Conclusions

There are a limited number of studies assessing the role of prophylactic pelvic drainage exclusively in patients with extraperitoneal rectal and anal anastomoses. Existing evidence does not support the hypothesis that prophylactic drainage may reduce the AL rate or improve patients’ outcome. Thus, their routine use cannot be justified. However, drain insertion does not appear to increase postoperative morbidity. In conclusion, although there is not sufficient evidence to support routine extraperitoneal anastomotic drainage, individualized patients’ management should also be considered in specific clinical scenarios. 
